# Clinical Manifestation of Cardiac Rupture in Patients with ST-Segment Elevation Myocardial Infarction: Early Versus Late Primary Percutaneous Coronary Intervention

**DOI:** 10.5334/gh.1155

**Published:** 2022-09-30

**Authors:** Xile Bi, Bin Wang, Gary Tse, Cuilian Dai, Xiang Chen, Fanqi Meng, Yan Wang

**Affiliations:** 1 Xiamen Cardiovescular Hospital Xiamen University, Xiamen, CN; 2 Cardiovascular Analytics Group, Laboratory of Cardiovascular Physiology, Hong Kong, CN

**Keywords:** cardiac rupture, Primary PCI, myocardial infarction

## Abstract

**Background:**

Cardiac rupture is one of the fatal complications of ST-Segment Elevation Myocardial Infarction (STEMI) in the primary percutaneous coronary intervention (PPCI) era. The present study aims to identify risk factors of cardiac rupture among patients suffering from STEMI, treated with early and late PPCI.

**Methods:**

This is a multicenter retrospective cohort study involving STEMI patients with cardiac rupture (CR group), matched with STEMI patients without CR (control group) in a 1:5 ratio. They were divided into the early (≤ 6 h) and the late (> 6 h) PCI groups. Multivariable logistic regression was utilized to identify risk factors for cardiac rupture.

**Results:**

Seventy-four patients in the CR and 370 in the control group were included. Multivariable regression identified lateral infarction (OR = 11.89, 95% CI 2.22–63.81, p < 0.01) in the early PCI phase as a significant risk factor for cardiac rupture. Thrombolysis in myocardial infarction (TIMI) grade 0-1 (early PCI: OR = 4.16, 95% CI 1.33-13.0, p = 0.01; late PCI: OR = 4.46, 95% CI 1.59–12.54, p < 0.01) was a risk factor for both early and late PCI groups. In contrast, TIMI grade 2 was associated with a higher rupture risk within the late (OR = 16.87, 95% CI 3.83–74.19, p < 0.001) but not for the early (OR = 5.44, 95% CI 0.76–39.07, p = 0.09) PCI groups. STEMI combined with Killip IV was associated with a higher rupture risk for the late PCI group (OR = 1.43, 95% CI 1.03–1.99, p = 0.04). Intra-aortic balloon pump (IABP) was protective against cardiac rupture within early PPCI (OR = 0.18, 95% CI 0.04–0.89, p = 0.04). In contrast, glycoprotein IIb/IIIa inhibitors were associated with lower rupture risks in both the early and late groups (early PCI: OR = 0.38, 95% CI 0.17–0.87, p = 0.02; late PCI: OR = 0.33, 95% CI 0.15–0.75, p < 0.01).

**Conclusions:**

No reflow or slow blood flow is associated with a higher risk of cardiac rupture in early and late PCI patients. Glycoprotein IIb/IIIa inhibitors are beneficial in preventing heart rupture, and the use of IABP in early PPCI is also helpful in preventing heart rupture.

## Introduction

Cardiac rupture is a fatal complication of ST-Segment Elevation Myocardial Infarction (STEMI). Although STEMI-related cardiac rupture has significantly fallen in the primary percutaneous coronary intervention (PPCI) era, it still accounts for one-third of the in-hospital death due to acute myocardial infarction (AMI). It is the second leading cause of death after heart failure [1]. Guidelines have recommended that the time window of PPCI for STEMI patients last up to 48 hours [2]. Despite efforts to reduce presentation time, approximately 30% of eligible patients did not receive PPCI therapy during the early stage due to the constraints of actual medical conditions and delayed transfer in many cases [3].

A delay in PPCI-related time could be a factor contributing to cardiac rupture. However, few studies have specifically explored the risk of cardiac rupture stratified by early versus late PPCI. Some investigators have raised new questions that reperfusion therapy may not reduce the early cardiac rupture incidence within STEMI and is only beneficial in late-onset cardiac rupture [4,5]. This study aims to compare the risk of cardiac rupture in patients with STEMI treated with early and late PPCI, to bring some enlightenment to our clinical intervention work.

## Methods

### Study population

The Ethics Committee of all the participating centers (Xiamen Cardiovascular Hospital Xiamen University; Peking University Third Hospital; Peking University First Hospital; The First Hospital of Qinhuangdao; Shanxi Cardiovascular Hospital; Yancheng First Hospital Affiliated of Nanjing University Medical School) approved the study. The current study was registered at ClinicalTrials.gov (NCT04172168).

This retrospective case-control study recruited consecutive cases of cardiac rupture and STEMI patients ohf six hospitals in China between January 2011 and December 2019. They all were STEMI patients who underwent PPCI combined with fatal left ventricular free wall rupture. In brief, patients were eligible for enrollment in the study if they had continuous typical chest pain of myocardial ischemia correlated with ST elevation of 0.1 mV in 2 adjacent electrocardiographic leads or complete left bundle branch block. Patients were excluded from the study if they had an aortic dissection, acute cerebrovascular disease, acute non-ST segment elevation myocardial infarction, ventricular septal rupture, ischemic mitral regurgitation, thrombolytic therapy, elective PCI, only coronary angiography (CAG) and conservative therapy. The PCI time was the interval from the onset of chest pain to the first balloon dilated (S2B). They were divided into two groups according to the 6-h boundary of the PPCI time window, which was defined as the early PCI group (≤6 h) and the late PCI group (>6 h). The control group was matched in a 1:5 ratio based on the age and gender of patients within the CR group. The matched patients were chosen from the central database. Seventy-four patients from the CR group and 370 from the control group were included (180 cases in the early and 190 in the late PCI control groups).

CR diagnostic criteria: based on the diagnosis of acute myocardial infarction, the symptoms, and signs of pericardial tamponade, bradycardia, escape and electromechanical separation on electrocardiogram (ECG), and pericardial effusion (at least 5 mm in the chest or under the sternum) on echocardiography. CR was not diagnosed if coronary artery perforation led to cardiac tamponade during PCI. The baseline data of the STEMI patients, including clinical characteristics, 18 lead ECG, echocardiography, laboratory results, and interventional procedure data, were recorded by designated personnel from each central hospital. The study conformed to the Declaration of Helsinki. Patients and the public were not involved in the research design, conduct, reporting, or dissemination.

Based on the CAG results, the following interventions were performed: drug-eluting stent implantation, percutaneous transluminal coronary angioplasty (PTCA), thrombus aspiration, and intra-aortic balloon pump or temporary pacemaker were performed. Patients also received a conventional oral loading dose of dual antiplatelet therapy before PPCI, and the activated clotting time (ACT) was maintained beyond 280 seconds during PPCI. If there was no contraindication after PPCI, dual antiplatelet therapy was routinely given. Except for coronary high load thrombosis, conventional anticoagulant therapy was not recommended. The interventional physician decided on using glycoprotein IIb/IIIa inhibitors during or after the operation. Additional medications for secondary prevention, including statins, β-blockers, and angiotensin-converting enzyme inhibitors, were prescribed based on current guidelines. The cardiogenic shock was defined as Killip IV grade. The analyses were performed independently by two experienced interventional cardiologists blinded to other parameters of patients and their clinical outcomes, and coronary flow was recorded with TIMI grading. The TIMI grading is defined as absent flow (TIMI flow grade 0), incomplete filling (TIMI flow grade 1), slow-reflow but complete filling (TIMI 2), or complete filling (TIMI 3) of the culprit coronary artery during or at the end of PPCI as revealed with the coronary angiogram.

### Statistical Analysis

The data were analyzed using SPSS version 22.0 (SPSS, Chicago, Ill, USA). Categorical variables as numbers and percentages and then using the X^2^ test. Continuous variables are presented as means±standard deviation (SD) or medians (interquartile range). The means of normally distributed variables were compared with the Student t-test. The distributions of skewed variables were compared with the Wilcoxon rank-sum test. The control group was matched in a 1:5 ratio based on the age and gender of patients in the CR group. We use 1:5 control group matching to make the test efficiency reach 80%. The multivariable analysis included all variables potentially related to CR risk to identify risk factors and the characteristics of coronary artery lesions for CR. The multivariable logistic regression (LR) models were constructed using the clinical baseline and coronary artery lesions variables to identify the risk factors for cardiac rupture after early and late PCI. The odds ratio (OR), 95% confidence interval (CI), and P-value were presented. Differences with a P-value of less than 0.05 were statistically significant.

## Results

### Study population and baseline clinical characteristic

**Figure 1:**
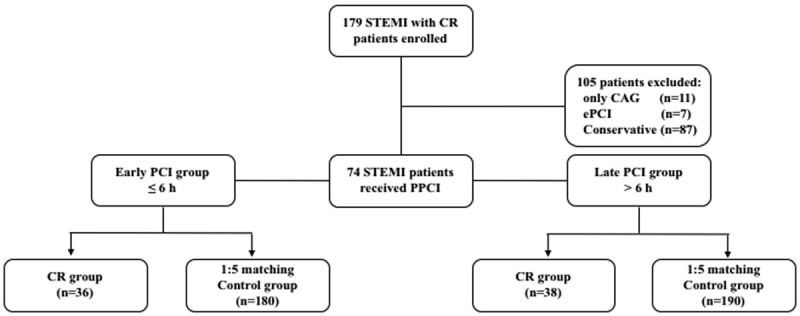
Trial profile.

A total of 179 patients with cardiac rupture after STEMI were included. The exclusion criteria were elective PCI (n = 7), only CAG (n = 11), and only the conservative group (n = 87). Finally, 74 patients with cardiac rupture after being treated using PPCI fulfilled the inclusion criteria (Figure 1). The baseline demographics, medical history, performance characteristics, and procedure data of STEMI patients in the early and late PCI groups are depicted in Tables 1 and 2. The univariable analysis of clinical characteristics with the risk of early and late PCI groups is represented in Supplementary Table 1. Of the 74 patients, 25.7% showed rupture within the first hour, and 58.1% of patients showed rupture within 24 hours of PPCI (Supplementary Figure 1). The results indicate no statistical difference between CR time and PPCI time during the early and late PCI stages (Supplementary Figure 2, p = 0.39).

**Table 1:** Baseline Clinical Characteristics.


	EARLY PCI			LATE PCI		
	
	CR	CONTROL	P VALUE	CR	CONTROL	P VALUE

Patients	36	180		38	190	

Age (years)	75 (67–78)	74 (67–78)	0.95	74 (65–79)	74 (65–79)	0.98

Stratify age (years)			1.0			1.0

< 60	5 (13.9)	25 (13.9)		2 (5.3)	10 (5.3)	

60–69	6 (16.7)	30 (16.7)		15 (39.5)	75 (39.5)	

70–79	22 (61.1)	110 (61.1)		13 (34.2)	65 (34.2)	

> 80	3 (8.3)	15 (8.3)		8 (21.1)	40 (21.1)	

Female	14 (38.9)	70 (38.9)	1.0	18 (47.4)	90 (47.4)	1.0

S2B time, h	3.9 (2.8–4.7)	4.1 (2.8–5.1)	0.48	9.9 (7.8–15.0)	11.7 (7.7–21.2)	0.31

**Medical history**						

Hypertension	21 (58.3)	99 (55.0)	0.71	27 (71.1)	107 (56.3)	0.10

Diabetes	14 (38.9)	51 (28.3)	0.21	12 (31.6)	47 (24.7)	0.38

Dyslipidemia	5 (13.9)	53 (29.4)	0.06	7 (18.4)	53 (27.9)	0.23

Smoking	11 (30.6)	73 (40.6)	0.26	18 (47.4)	71 (37.4)	0.25

Renal insufficiency	5 (13.9)	20 (11.1)	0.64	7 (18.4)	24 (12.6)	0.35

Prior CVD	3 (8.3)	5 (2.8)	0.13	2 (5.3)	7 (3.7)	0.65

Prior MI	0 (0)	8 (4.4)	1.0	1 (2.6)	5 (2.6)	1.0

Prior CABG	0 (0)	0 (0)	1.0	1 (2.6)	1 (0.5)	0.25

IHD	2 (5.6)	20 (11.1)	0.33	3 (7.9)	22 (11.6)	0.51

Killip class IV	4 (11.1)	19 (10.6)	0.92	7 (18.4)	7 (3.7)	0.002

**STEMI Location**			0.006			0.07

Inferior	12 (33.3)	90 (50.0)		12 (31.6)	84 (44.2)	

Anterior	19 (52.8)	87 (48.3)		22 (57.9)	100 (52.6)	

Lateral	5 (13.9)	3 (1.7)		4 (10.5)	6 (3.2)	

**Treatment at first 24 h**						

Asprin	36 (100)	177 (98.3)	0.99	38 (100)	189 (99.5)	0.37

Clopidogrel/Tigorila	36 (100)	178 (98.9)	0.74	37 (97.4)	190 (100)	0.37

Beta-blocker	18 (50.0)	137 (76.1)	0.003	15 (39.5)	140 (73.7)	< 0.001

Statin	33 (91.7)	173 (96.1)	0.47	36 (94.7)	186 (97.9)	0.58

ACEI/ARB	15 (41.7)	130 (72.2)	< 0.001	14 (36.8)	135 (71.1)	< 0.001

Calcium antagonist	4 (11.1)	8 (4.4)	0.23	3 (7.9)	32 (16.8)	0.25

LMWH	6 (16.7)	44 (24.4)	0.43	10 (26.3)	73 (38.4)	0.22


CR, cardiac rupture; CVD, cerebrovascular disease; MI, myocardial infarction; CABG, coronary artery bypass graft; S2B, symptom to balloon. IHD, schemic heart disease.

**Table 2:** Procedural and Lesion characteristics.


	EARLY PCI			LATE PCI		

	CR	CONTROL	P VALUE	CR	CONTROL	P VALUE

Patients	36	180		38	190	

**procedures**						

IABP	2 (5.6)	34 (18.9)	0.07	5 (13.2)	26 (13.7)	0.93

Pacemaker	6 (16.7)	28 (15.6)	0.87	4 (10.5)	20 (10.5)	1.0

Thrombus aspiration	14 (38.9)	101 (56.1)	0.06	12 (31.6)	81 (42.6)	0.21

GPI	10 (27.8)	101 (56.1)	0.02	12 (31.6)	110 (57.9)	0.004

Malignant arrhythmia	8 (22.2)	21 (11.7)	0.10	7 (18.4)	12 (6.3)	0.02

**Lesion location**			0.39			0.21

LM	0 (0)	5 (2.8)		1 (2.6)	1 (0.5)	

LAD	19 (52.8)	82 (45.6)		19 (50.0)	98 (51.6)	

LCX	6 (16.7)	16 (8.9)		5 (13.2)	24 (12.6)	

RCA	11 (30.6)	75 (41.7)		11 (28.9)	65 (34.2)	

Diagonal	0 (0)	2 (1.1)		2 (5.3)	2 (1.1)	

**No. diseased vessel**			0.14			0.17

One-vessel disease	11 (30.6)	83 (46.1)		10 (26.3)	81 (42.6)	

Two-vessel disease	8 (22.2)	57 (31.7)		15 (39.5)	60 (31.6)	

Three-vessel disease	17 (47.2)	40 (22.2)		13 (34.2)	49 (25.8)	

**LM disease**	4 (11.1)	18 (10.0)	0.84	5 (13.2)	22 (11.6)	0.78

**Lesion segment**			0.48			0.06

Proximal	23 (63.9)	103 (57.2)		27 (71.0)	96 (50.5)	

Middle	8 (22.2)	58 (32.2)		8 (21.1)	59 (31.1)	

Distal	5 (13.9)	19 (10.6)		3 (7.9)	35 (18.4)	

**Initial TIMI grade**			0.46			0.49

0–1	35 (97.2)	151 (83.9)		36 (94.7)	144 (75.8)	

2	0 (0)	13 (7.2)		2 (5.3)	20 (10.5)	

3	1 (2.8)	16 (8.9)		0 (0)	26 (13.7)	

**Final TIMI grade**			0.02			< 0.001

0–1	3 (8.3)	3 (1.7)		8 (21.1)	3 (1.6)	

2	6 (16.7)	12 (6.7)		8 (21.1)	15 (7.9)	

3	27 (75)	165 (91.7)		22 (57.8)	172 (90.5)	


Data are shown as n (%); CR, cardiac rupture; IABP, Intra-aortic balloon pump; GPI, Glycoprotein IIb/IIIa inhibitor; LM, left main; LAD, left anterior descending; LCX, left circumflex; RCA, right coronary; TIMI, thrombolysis in myocardial infarction.

### Multivariable analysis

Multivariable logistic regression identified lateral infarction as a significant risk factor for cardiac rupture in the early PPCI group (OR = 11.89, 95%CI 2.22–63.81, p < 0.01). TIMI grade 0–1 was related to a higher rupture risk during early (OR = 4.16, 95% CI 1.33–13.0, p = 0.01) and late (OR = 4.46, 95% CI 1.59–12.54, p < 0.01) PPCI. TIMI grade 2 was associated with a higher rupture risk during late PPCI (OR = 16.87, 95% CI 3.83–74.19, p < 0.001) but not during the early PCI phase (OR = 5.44, 95% CI 0.76–39.07, p = 0.09). STEMI combined with Killip IV was related to a higher rupture risk in the late PCI group (OR = 1.43, 95% CI 1.03–1.99, p = 0.04). IABP was protective against cardiac rupture during early PPCI (OR = 0.18, 95% CI 0.04–0.89, p = 0.04; Figure 2A). The use of glycoprotein IIb/IIIa inhibitors was protective against cardiac rupture in early (OR = 0.38, 95% CI 0.17–0.87, p = 0.02 and late PPCI (OR = 0.33, 95% CI 0.15–0.75, p < 0.01; Figure 2A and B).

**Figure 2A:**
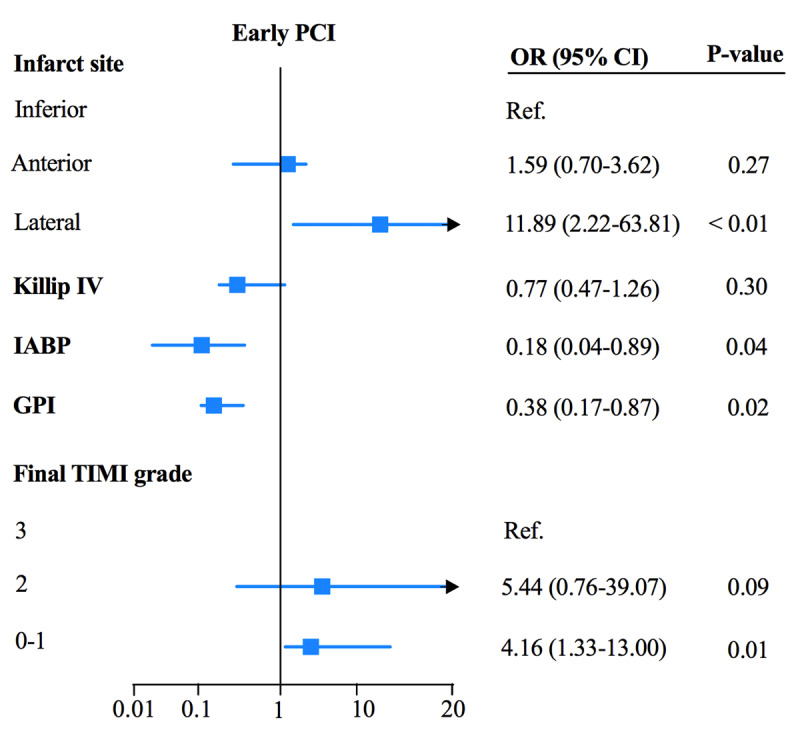
Factors related to CR with early PCI within the multivariable model.

**Figure 2B:**
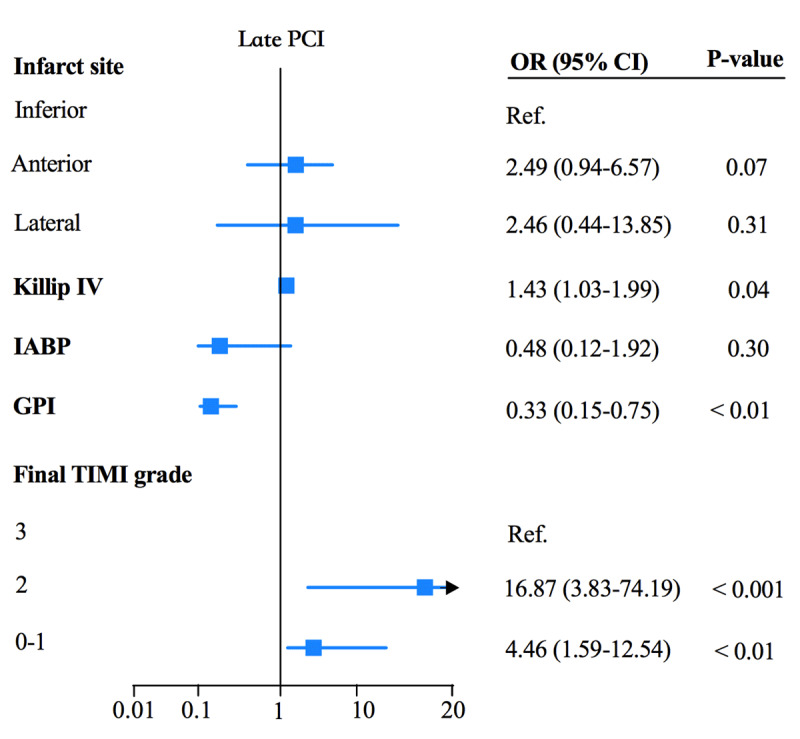
Factors associated with CR with late PCI within the multivariable model.

## Discussion

This is the first study to compare the risk of cardiac rupture in patients undergoing early PPCI (less than 6 hours) and late PPCI (6–72 hours) for STEMI to the best of our knowledge. The main findings are that (1) cardiac rupture is more prone to occur in lateral STEMI patients who received early PPCI. (2) No reflow or slow blood flow was associated with higher rupture risk, irrespective of PPCI timing. (3) Glycoprotein IIb/IIIa inhibitors are beneficial to the prevention of heart rupture, and IABP use in early PPCI is also beneficial to the prevention of heart rupture, and (4) STEMI with cardiogenic shock is significantly suspicious of cardiac rupture.

### Risk of cardiac rupture in early and late PPCI

STEMI within 12 hours of onset is a class I indication of PPCI, and STEMI within 12–48 hours of onset is a class IIa PPCI indication. The current study considered that defining the time from chest pain symptoms to the first balloon dilated time with a cut-off of 6 hours was a relatively early setting for comparing early and late PCI. Identifying a precise PPCI time frame as the point of inflection at which high-risk factors for cardiac rupture change was challenging. There has been a progressive decline in the incidence of CR over the past few decades. This is likely multifactorial, attributable to better therapeutic options for improving revascularization and reducing infarct size [6]. Our study found that no flow or slow blood flow was related to higher risks of cardiac rupture, regardless of the reperfusion timing. Previous studies have reported that the absence of reflow or continuous reperfusion injury during operation may be associated with higher risks of cardiac rupture [7,8,9,10,11]. In the no-reflow phenomenon, reperfusion injury results in myocyte damage through myocardial stunning, microvascular, and endothelial injury [12]. Therefore, we should be cautious in treating patients with STEMI demonstrating severe angiographic no-reflow or slow blood flow phenomenon. The study found that using glycoprotein IIb/IIIa inhibitors was protective against cardiac rupture, explaining the reduction in no-reflow or slow blood flow, in keeping with previous findings [13]. Theoretically, using an IABP could increase coronary perfusion and cardiac output, reduce afterload, decrease myocardial oxygen consumption, and improve hemodynamics. This study found that in patients with early PPCI, IABP could reduce heart rupture after STEMI. First, IABP may reduce mortality and improve prognosis in AMI patients having cardiogenic shock [14]. Meanwhile, STEMI with cardiogenic shock is highly suspicious of cardiac rupture, especially during the initial stage of subacute cardiac rupture. It is easy to be obscured by the appearance of cardiogenic shock [15]. Secondly, based on drugs such as verapamil, adenosine, and sodium nitroprusside, once the no-reflow phenomenon is established. It is a common clinical practice to use IABP in patients having a no-reflow phenomenon with the expectation of an increase in diastolic coronary flow and enhanced cardiac function [16]. However, its clinical value in STEMI patient population with the no-reflow or cardiogenic shock remains controversial [17,18]. Although thrombus aspiration devices were not associated with a lower incidence of cardiac rupture, they should be considered in selected cases [19]. Therefore, the combination of glycoprotein IIb/IIIa inhibitors and IABP devices in limiting reperfusion injury could provide the best clinical benefit.

### Mechanisms of rupture in the PPCI for STEMI

Lateral infarction was significantly related to increased risks of rupture in the early PPCI group but not in the late PPCI group. In addition to reperfusion injury, mechanical factors may play essential roles in the pathogenesis of cardiac rupture. Takada et al. revealed that endocardial tears of lateral infarction concentrate on two sites: at or near the base of the papillary muscles and in the area where the septum meets the cardiac free wall [20]. The rupture could be associated with papillary muscles at higher tensile strength sites [21].

The most cardiac rupture occurred within 24 hours of PPCI (58.1%), with around 25.7% of cases occurring within the first hour. The early rupture was also observed in the APEX-AMI trial (The mean time of onset of complications is 20.7 hours) [22]. Meanwhile, the TIMI II [23] and NRMI study [24] also observed that the timing of onset of cardiac rupture also occurs earlier in the course of STEMI when receiving thrombolytic therapy than during the reperfusion era. A vital issue brought by these two reperfusion treatments is the no-reflow phenomenon and reperfusion injury to the potential risk of cardiac rupture. The reperfusion therapy decreases the overall risk of myocardial rupture by reducing the extent and transmurality of myocardial necrosis. However, it may elevate early risk due to intramyocardial hemorrhage. Honda et al. found that patients with PPCI or thrombolysis suffered a similar risk of intramyocardial hemorrhage (the myocardial hemorrhage rate was 83% with PPCI, compared with 71% for thrombolysis and 18% without reperfusion therapy) [6]. Ganame J et al. observed that myocardial hemorrhage is present in at least 25% of patients in the early stage after PPCI using cardiac magnetic resonance imaging (MRI) [25]. Intramyocardial hemorrhage could occur as part of reperfusion injury. Intramyocardial bleeding causes dissection of the infarcted myocardium and delays the healing process. Adverse effects on left ventricular remodeling may increase the risk of rupture in Becker type I and II [6,26,27,28,29,30]. The risk of myocardial hemorrhage may be attributed to reperfusion therapy as a unique complication. The evolution of myocardial infarction (including Q wave time shift forward, ST-segment elevation, and creatine kinase index) was also probably a contributing factor to cardiac rupture [5]. Finally, even though more than half of cardiac ruptures occur within 24 h after PPCI, the apparent survival advantage of PPCI treatment cannot be concealed [31]. In our study, 41.9% of patients had cardiac rupture 24 hours after PPCI. The initial development of rupture may not be rapid. It may be related to slow tear progression in areas of myocardial erosion, incomplete myocardial tear, or epicardial fibrin deposit formation with adhesion to the myocardium [32,33,34]. This study describes the different characteristics of cardiac rupture after early and late PPCI and hopes to be helpful to the individualized PPCI program.

Several limitations of this study should be recognized. First, this sample was biased because of the PPCI design requirement. The incidence of CR would be lower in the STEMI population of patients, and some patients admitted for STEMI complicated CR did not have the opportunity to receive PPCI. Retrospective studies bear the limitations of missing data, selection bias, and unexplained confounding factors. Second, the number in some subgroups samples was relatively small due to the low prevalence of cardiac rupture. Third, Some of the patients may present with CR at admission. The medical treatments including beta-blockers and ACEI/ARB have applied limited base on the Killip grading III-IV. However, most medication uses did not differ in patients between early and late PPCI, which will be an extra confounding factor for detailed analysis of survival predictors. Fourth, this study did not assess the impact of complete revascularization on CR. Fifth, some data may have been confounding due to the prolonged research time frame from 2011 to 2019, and the field of coronary heart disease has changed rapidly during that time.

Therefore, no reflow or slow blood flow is associated with a higher risk of cardiac rupture in early and late PCI patients. Glycoprotein IIb/IIIa inhibitors could prevent heart rupture, and the use of IABP in early PPCI is also advantageous to the prevention of heart rupture

## Additional File

The additional file for this article can be found as follows:

10.5334/gh.1155.s1 Supplementary Files. Supplementary Figures 1, 2 and Table 1.
