# Supplementary material for: Clinical Manifestation of Cardiac Rupture in Patients with ST-Segment Elevation Myocardial Infarction: Early Versus Late Primary Percutaneous Coronary Intervention
Source: Glob Heart. 2022 Sep 30;17(1):69. doi: 10.5334/gh.1155 (PMC9524297; doi:10.5334/gh.1155)
Supplement: Supplementary Files. — Supplementary Figures 1, 2 and Table 1. [file gh-17-1-1155-s1.zip › gh-17-1-1155-s1/Supplementary Table 1.docx]

**Supplementary Table 1.** The univariable analysis of clinical characteristics with the risk of early and late PCI groups

| **Variables** | **Early PCI** | | | | **Late PCI** | | | |
| --- | --- | --- | --- | --- | --- | --- | --- | --- |
|  | **CR (n=36)** | **Control (n=180)** | **OR (95%CI)** | **p Value** | **CR (n=38)** | **Control (n=190)** | **OR (95%CI)** | **p Value** |
| **Clinical diagnosis** |  |  |  | 0.006 |  |  |  | 0.07 |
| Inferior | 12 (33.3) | 90 (50.0) | Ref. |  | 12 (31.6) | 84 (44.2) | Ref. |  |
| Anterior | 19 (52.8) | 87 (48.3) | 1.64 (0.75-3.58) |  | 22 (57.9) | 100 (52.6) | 1.54 (0.72-3.30) |  |
| Lateral | 5 (13.9) | 3 (1.7) | 12.5 (2.65-59.07) |  | 4 (10.5) | 6 (3.2) | 4.67 (1.15-18.97) |  |
| **Hypertension** |  |  |  | 0.71 |  |  |  |  |
| No | 15 (41.7) | 81 (45.0) | Ref. |  | 11 (28.9) | 83 (43.7) | Ref. |  |
| Yes | 21 (58.3) | 99 (55.0) | 1.15 (0.56-2.36) |  | 27 (71.1) | 107 (56.3) | 1.90 (0.89-4.06) |  |
| **Diabetes** |  |  |  | 0.21 |  |  |  | 0.38 |
| No | 22 (61.1) | 129 (71.7) | Ref. |  | 26 (68.4) | 143 (75.3) | Ref. |  |
| Yes | 14 (38.9) | 51 (28.3) | 1.61 (0.77-3.39) |  | 12 (31.6) | 47 (24.7) | 1.40 (0.66-3.00) |  |
| **Dyslipidemia** |  |  |  | 0.06 |  |  |  | 0.23 |
| No | 31 (86.1) | 127 (70.6) | Ref. |  | 31 (81.6) | 137 (72.1) | Ref. |  |
| Yes | 5 (13.9) | 53 (29.4) | 0.39 (0.14-1.05) |  | 7 (18.4) | 53 (27.9) | 0.58 (0.24-1.41) |  |
| **Smoking** |  |  |  | 0.26 |  |  |  | 0.25 |
| No | 25 (69.4) | 107 (59.4) | Ref. |  | 20 (52.6) | 119 (62.6) | Ref. |  |
| Yes | 11 (30.6) | 73 (40.6) | 0.65 (0.30-1.39) |  | 18 (47.4) | 71 (37.4) | 1.51 (0.75-3.04) |  |
| **Prior CVD** |  |  |  | 0.13 |  |  |  | 0.65 |
| No | 33 (91.7) | 175 (97.2) | Ref. |  | 36 (94.7) | 183 (96.3) | Ref. |  |
| Yes | 3 (8.3) | 5 (2.8) | 3.18 (0.73-13.96) |  | 2 (5.3) | 7 (3.7) | 1.45 (0.29-7.28) |  |
| **IHD** |  |  |  | 0.33 |  |  |  | 0.51 |
| No | 34 (94.4) | 160 (88.9) | Ref. |  | 35 (92.1) | 168 (88.4) | Ref. |  |
| Yes | 2 (5.6) | 20 (11.1) | 0.47 (0.11-2.11) |  | 3 (7.9) | 22 (11.6) | 0.66 (0.19-2.31) |  |
| **Killip class IV** |  |  |  | 0.92 |  |  |  |  |
| No | 32 (88.9) | 161 (89.4) | Ref. |  | 31 (81.6) | 183 (96.3) | Ref. |  |
| Yes | 4 (11.1) | 19 (10.6) | 1.01 (0.76-1.35) |  | 7 (18.4) | 7 (3.7) | 1.56 (1.18-2.06) |  |
| **IABP** |  |  |  | 0.07 |  |  |  | 0.93 |
| No | 34 (94.4) | 146 (81.1) | Ref. |  | 33 (86.8) | 164 (86.3) | Ref. |  |
| Yes | 2 (5.6) | 34 (18.9) | 0.25 (0.06-1.10) |  | 5 (13.2) | 26 (13.7) | 0.96 (0.34-2.67) |  |
| **Pacemaker** |  |  |  | 0.87 |  |  |  | 1.0 |
| No | 30 (83.3) | 152 (84.4) | Ref. |  | 34 (89.5) | 170 (89.5) | Ref. |  |
| Yes | 6 (16.7) | 28 (15.6) | 1.09 (0.41-2.85) |  | 4 (10.5) | 20 (10.5) | 1.00 (0.32-3.11) |  |
| **Thrombus aspiration** |  |  |  | 0.06 |  |  |  | 0.21 |
| No | 22 (61.1) | 79 (43.9) | Ref. |  | 26 (68.4) | 109 (57.4) | Ref. |  |
| Yes | 14 (38.9) | 101 (56.1) | 0.50 (0.24-1.04) |  | 12 (31.6) | 81 (42.6) | 0.62 (0.30-1.30) |  |
| **GPI** |  |  |  | 0.02 |  |  |  | 0.004 |
| No | 26 (72.2) | 79 (43.9) | Ref. |  | 26 (68.4) | 80 (42.1) | Ref. |  |
| Yes | 10 (27.8) | 101 (56.1) | 0.39 (0.18-0.83) |  | 12 (31.6) | 110 (57.9) | 0.34 (0.16-0.71) |  |
| **Malignant arrhythmia** |  |  |  | 0.10 |  |  |  | 0.02 |
| No | 28 (77.8) | 159 (88.3) | Ref. |  | 31 (81.6) | 178 (93.7) | Ref. |  |
| Yes | 8 (22.2) | 21 (11.7) | 2.16 (0.87-5.36) |  | 7 (18.4) | 12 (6.3) | 3.35 (1.22-9.17) |  |
| **No. diseased vessel** |  |  |  | 0.14 |  |  |  | 0.17 |
| One-vessel disease | 11 (30.6) | 83 (46.1) | Ref. |  | 10 (26.3) | 81 (42.6) | Ref. |  |
| Two-vessel disease | 8 (22.2) | 57 (31.7) | 1.59 (0.66-3.85) |  | 15 (39.5) | 60 (31.6) | 2.03 (0.85-4.82) |  |
| Three-vessel disease | 17 (47.2) | 40 (22.2) | 2.45 (1.01-5.96) |  | 13 (34.2) | 49 (25.8) | 2.15 (0.88-5.27) |  |
| **LM disease** |  |  |  | 0.84 |  |  |  | 0.78 |
| No | 32 (88.9) | 162 (90.0) | Ref. |  | 33 (86.8) | 168 (88.4) | Ref. |  |
| Yes | 4 (11.1) | 18 (10.0) | 1.13 (0.36-3.55) |  | 5 (13.2) | 22 (11.6) | 1.16 (0.41-3.27) |  |
| **Lesion segment** |  |  |  | 0.48 |  |  |  | 0.06 |
| Proximal | 23 (63.9) | 103 (57.2) | Ref. |  | 27 (71.0) | 96 (50.5) | Ref. |  |
| Middle | 8 (22.2) | 58 (32.2) | 1.91 (0.56-6.54) |  | 8 (21.1) | 59 (31.1) | 0.63 (0.15-2.54) |  |
| Distal | 5 (13.9) | 19 (10.6) | 1.62 (0.68-3.85) |  | 3 (7.9) | 35 (18.4) | 2.07 (0.88-4.87) |  |
| **Final TIMI grade** |  |  |  | 0.02 |  |  |  | <0.001 |
| 3 | 27 (75) | 165 (91.7) | Ref. |  | 22 (57.8) | 172 (90.5) | Ref. |  |
| 2 | 6 (16.7) | 12 (6.7) | 6.11 (1.17-31.86) |  | 8 (21.1) | 15 (7.9) | 20.85 (5.15-84.47) |  |
| 0-1 | 3 (8.3) | 3 (1.7) | 3.06 (1.06-8.83) |  | 8 (21.1) | 3 (1.6) | 4.17 (1.59-10.96) |  |

Datas are shown as n (%); OR, odds ratio; CR, cardiac rupture; IHD, schemic heart disease; IABP, Intra-aortic balloon pump; GPI, Glycoprotein IIb/IIIa inhibitor; LM, left main; LAD, left anterior descending; LCX, left circumflex; RCA, right coronary; TIMI, thrombolysis in myocardial infarction.
