# Supplementary material for: Clinical Manifestation of Cardiac Rupture in Patients with ST-Segment Elevation Myocardial Infarction: Early Versus Late Primary Percutaneous Coronary Intervention
Source: Glob Heart. 2022 Sep 30;17(1):69. doi: 10.5334/gh.1155 (PMC9524297; doi:10.5334/gh.1155)
Supplement: Supplementary Files. — Supplementary Figures 1, 2 and Table 1. [file gh-17-1-1155-s1.zip › gh-17-1-1155-s1/Supplementary Table 1.pdf]

Supplementary Table 1. The univariable analysis of clinical characteristics with the risk of early and late PCI groups

Variables	Early PCI				Late PCI			
	**CR (n=36)**	**Control (n=180)**	**OR (95%CI)**	**p Value**	**CR (n=38)**	**Control (n=190)**	**OR (95%CI)**	**p Value**
**Clinical diagnosis**				0.006				0.07
Inferior	12 (33.3)	90 (50.0)	Ref.		12 (31.6)	84 (44.2)	Ref.	
Anterior	19 (52.8)	87 (48.3)	1.64 (0.75-3.58)		22 (57.9)	100 (52.6)	1.54 (0.72-3.30)	
Lateral	5 (13.9)	3 (1.7)	12.5 (2.65-59.07)		4 (10.5)	6 (3.2)	4.67 (1.15-18.97)	
**Hypertension**				0.71				
No	15 (41.7)	81 (45.0)	Ref.		11 (28.9)	83 (43.7)	Ref.	
Yes	21 (58.3)	99 (55.0)	1.15 (0.56-2.36)		27 (71.1)	107 (56.3)	1.90 (0.89-4.06)	
**Diabetes**				0.21				0.38
No	22 (61.1)	129 (71.7)	Ref.		26 (68.4)	143 (75.3)	Ref.	
Yes	14 (38.9)	51 (28.3)	1.61 (0.77-3.39)		12 (31.6)	47 (24.7)	1.40 (0.66-3.00)	
**Dyslipidemia**				0.06				0.23
No	31 (86.1)	127 (70.6)	Ref.		31 (81.6)	137 (72.1)	Ref.	
Yes	5 (13.9)	53 (29.4)	0.39 (0.14-1.05)		7 (18.4)	53 (27.9)	0.58 (0.24-1.41)	

**Smoking**				0.26				0.25
No	25 (69.4)	107 (59.4)	Ref.		20 (52.6)	119 (62.6)	Ref.	
Yes	11 (30.6)	73 (40.6)	0.65 (0.30-1.39)		18 (47.4)	71 (37.4)	1.51 (0.75-3.04)	
**Prior CVD**				0.13				0.65
No	33 (91.7)	175 (97.2)	Ref.		36 (94.7)	183 (96.3)	Ref.	
Yes	3 (8.3)	5 (2.8)	3.18 (0.73-13.96)		2 (5.3)	7 (3.7)	1.45 (0.29-7.28)	
**IHD**				0.33				0.51
No	34 (94.4)	160 (88.9)	Ref.		35 (92.1)	168 (88.4)	Ref.	
Yes	2 (5.6)	20 (11.1)	0.47 (0.11-2.11)		3 (7.9)	22 (11.6)	0.66 (0.19-2.31)	
**Killip class IV**				0.92				
No	32 (88.9)	161 (89.4)	Ref.		31 (81.6)	183 (96.3)	Ref.	
Yes	4 (11.1)	19 (10.6)	1.01 (0.76-1.35)		7 (18.4)	7 (3.7)	1.56 (1.18-2.06)	
**IABP**				0.07				0.93
No	34 (94.4)	146 (81.1)	Ref.		33 (86.8)	164 (86.3)	Ref.	
Yes	2 (5.6)	34 (18.9)	0.25 (0.06-1.10)		5 (13.2)	26 (13.7)	0.96 (0.34-2.67)	
**Pacemaker**				0.87				1.0
No	30 (83.3)	152 (84.4)	Ref.		34 (89.5)	170 (89.5)	Ref.	

Yes	6 (16.7)	28 (15.6)	1.09 (0.41-2.85)		4 (10.5)	20 (10.5)	1.00 (0.32-3.11)	
**Thrombus aspiration**				0.06				0.21
No	22 (61.1)	79 (43.9)	Ref.		26 (68.4)	109 (57.4)	Ref.	
Yes	14 (38.9)	101 (56.1)	0.50 (0.24-1.04)		12 (31.6)	81 (42.6)	0.62 (0.30-1.30)	
**GPI**				0.02				0.004
No	26 (72.2)	79 (43.9)	Ref.		26 (68.4)	80 (42.1)	Ref.	
Yes	10 (27.8)	101 (56.1)	0.39 (0.18-0.83)		12 (31.6)	110 (57.9)	0.34 (0.16-0.71)	
**Malignant arrhythmia**				0.10				0.02
No	28 (77.8)	159 (88.3)	Ref.		31 (81.6)	178 (93.7)	Ref.	
Yes	8 (22.2)	21 (11.7)	2.16 (0.87-5.36)		7 (18.4)	12 (6.3)	3.35 (1.22-9.17)	
**No. diseased vessel**				0.14				0.17
One-vessel disease	11 (30.6)	83 (46.1)	Ref.		10 (26.3)	81 (42.6)	Ref.	
Two-vessel disease	8 (22.2)	57 (31.7)	1.59 (0.66-3.85)		15 (39.5)	60 (31.6)	2.03 (0.85-4.82)	
Three-vessel disease	17 (47.2)	40 (22.2)	2.45 (1.01-5.96)		13 (34.2)	49 (25.8)	2.15 (0.88-5.27)	
**LM disease**				0.84				0.78
No	32 (88.9)	162 (90.0)	Ref.		33 (86.8)	168 (88.4)	Ref.	

Yes	4 (11.1)	18 (10.0)	1.13 (0.36-3.55)		5 (13.2)	22 (11.6)	1.16 (0.41-3.27)	
**Lesion segment**				0.48				0.06
Proximal	23 (63.9)	103 (57.2)	Ref.		27 (71.0)	96 (50.5)	Ref.	
Middle	8 (22.2)	58 (32.2)	1.91 (0.56-6.54)		8 (21.1)	59 (31.1)	0.63 (0.15-2.54)	
Distal	5 (13.9)	19 (10.6)	1.62 (0.68-3.85)		3 (7.9)	35 (18.4)	2.07 (0.88-4.87)	
**Final TIMI grade**				0.02				<0.001
3	27 (75)	165 (91.7)	Ref.		22 (57.8)	172 (90.5)	Ref.	
2	6 (16.7)	12 (6.7)	6.11 (1.17-31.86)		8 (21.1)	15 (7.9)	20.85 (5.15-84.47)	
0-1	3 (8.3)	3 (1.7)	3.06 (1.06-8.83)		8 (21.1)	3 (1.6)	4.17 (1.59-10.96)	

Datas are shown as n (%); OR, odds ratio; CR, cardiac rupture; IHD, schemic heart disease; IABP, Intra-aortic balloon pump; GPI, Glycoprotein IIb/IIIa inhibitor; LM, left main; LAD, left anterior descending; LCX, left circumflex; RCA, right coronary; TIMI, thrombolysis in myocardial infarction.
