# Supplementary figures and images for: Clinical Manifestation of Cardiac Rupture in Patients with ST-Segment Elevation Myocardial Infarction: Early Versus Late Primary Percutaneous Coronary Intervention
Source: Glob Heart. 2022 Sep 30;17(1):69. doi: 10.5334/gh.1155 (PMC9524297; doi:10.5334/gh.1155)

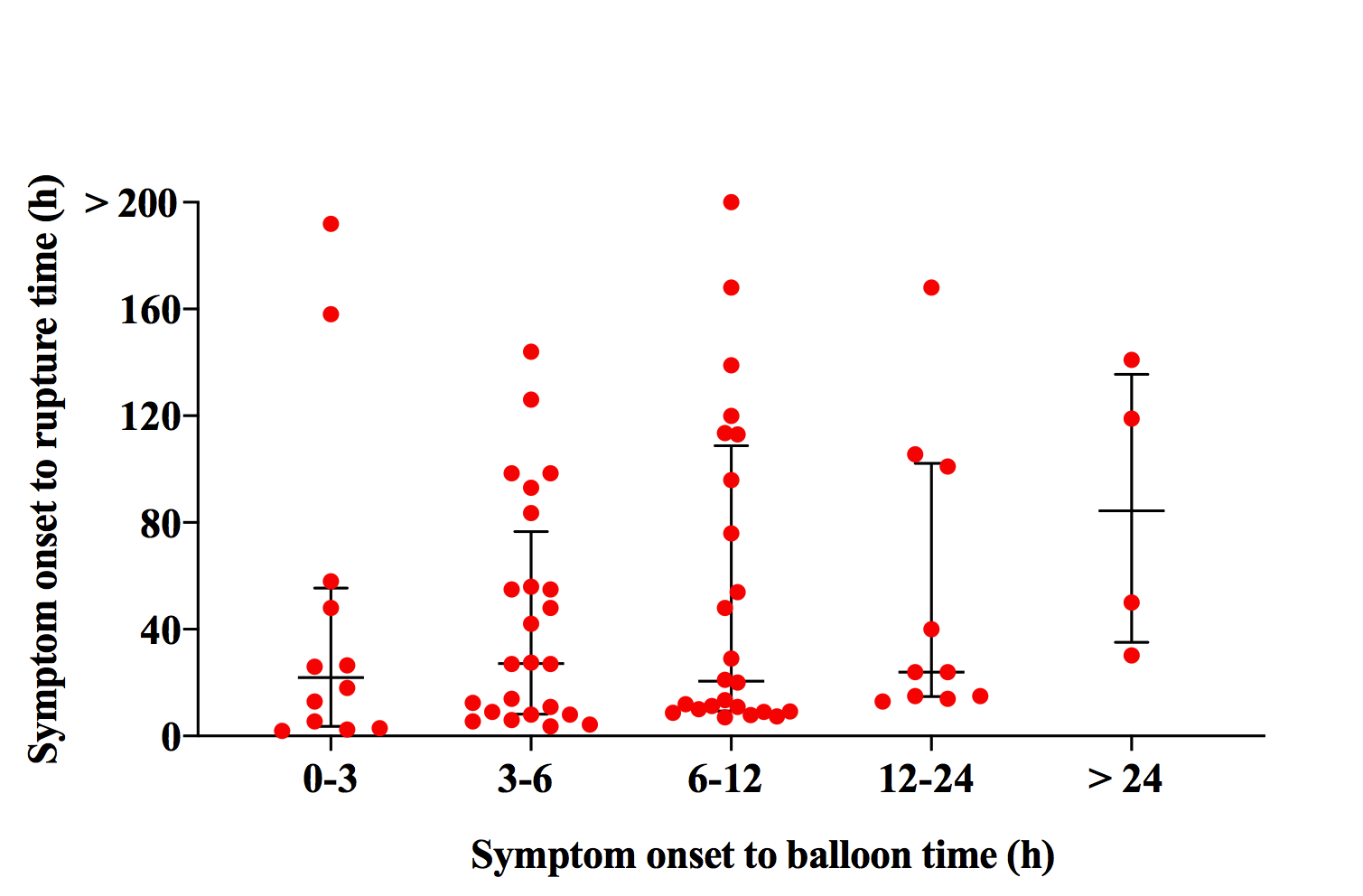

Supplement: Supplementary Files. — Supplementary Figures 1, 2 and Table 1. [file gh-17-1-1155-s1.zip › gh-17-1-1155-s1/Supplementary Fig 1.tiff]

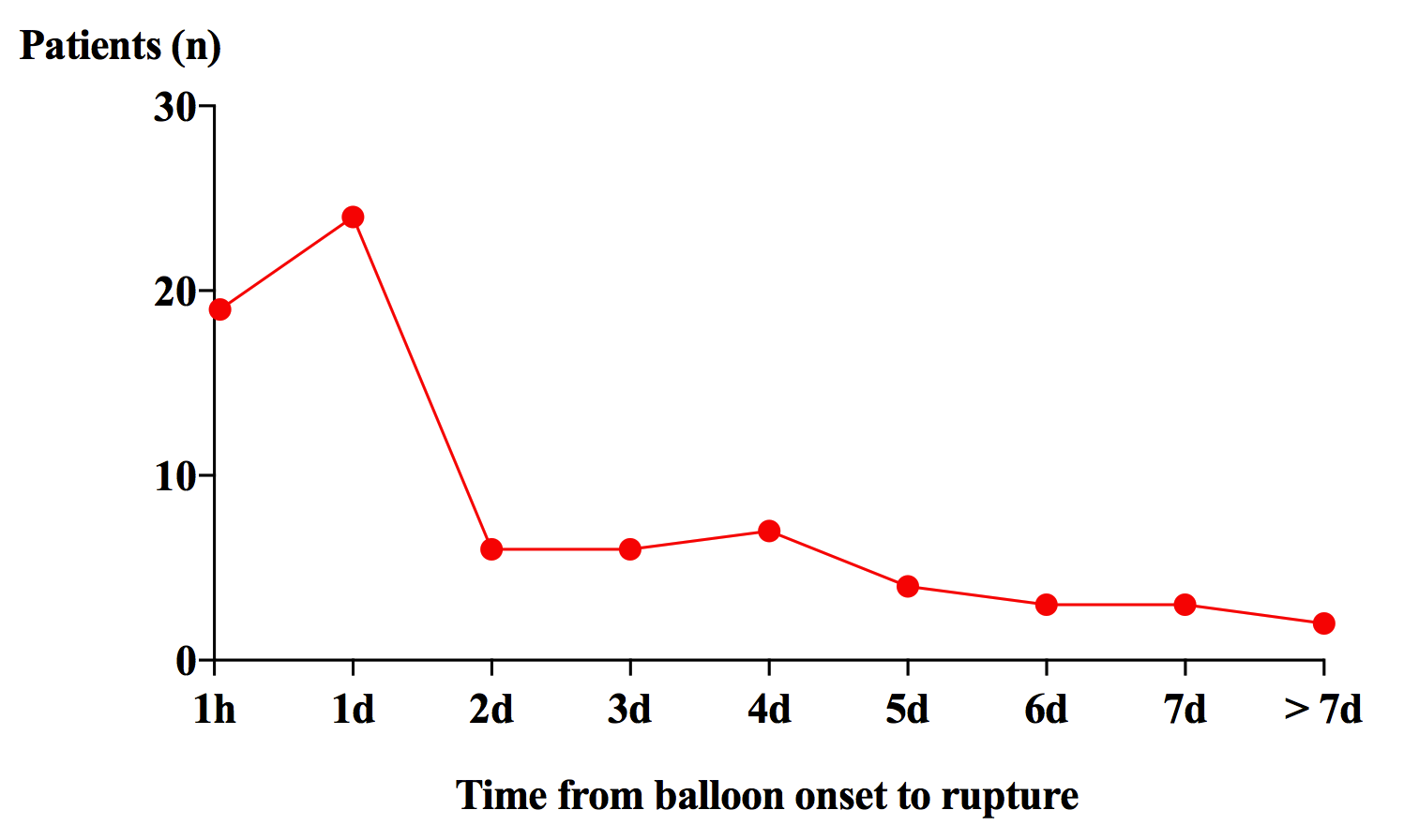

Supplement: Supplementary Files. — Supplementary Figures 1, 2 and Table 1. [file gh-17-1-1155-s1.zip › gh-17-1-1155-s1/Supplementary Fig 2.tiff]
